# Marine Carotenoids and Cardiovascular Risk Markers

**DOI:** 10.3390/md9071166

**Published:** 2011-06-27

**Authors:** Graziano Riccioni, Nicolantonio D’Orazio, Sara Franceschelli, Lorenza Speranza

**Affiliations:** 1 Cardiology Unit, San Camillo de Lellis Hospital, via Isonzo, Manfredonia, Foggia, 71043, Italy; 2 Human Nutrition, Department of Biomedical Science, via Dei vestini, University G. D’Annunzio, Chieti, 66013, Italy; E-Mail: ndorazio@unich.it; 3 Department of Human Movement Sciences, via Dei Vestini, University G. D’Annunzio, Chieti, 66013, Italy; E-Mails: s.francheschelli@unich.it (S.F.); l.speranza@unich.it (L.S.)

**Keywords:** marine carotenoids, astaxanthin, fucoxantin, atherosclerosis, cardiovascular disease, free radicals, oxidized LDL

## Abstract

Marine carotenoids are important bioactive compounds with physiological activities related to prevention of degenerative diseases found principally in plants, with potential antioxidant biological properties deriving from their chemical structure and interaction with biological membranes. They are substances with very special and remarkable properties that no other groups of substances possess and that form the basis of their many, varied functions and actions in all kinds of living organisms. The potential beneficial effects of marine carotenoids have been studied particularly in astaxanthin and fucoxanthin as they are the major marine carotenoids. Both these two carotenoids show strong antioxidant activity attributed to quenching singlet oxygen and scavenging free radicals. The potential role of these carotenoids as dietary anti-oxidants has been suggested to be one of the main mechanisms for their preventive effects against cancer and inflammatory diseases. The aim of this short review is to examine the published studies concerning the use of the two marine carotenoids, astaxanthin and fucoxanthin, in the prevention of cardiovascular diseases.

## 1. Introduction

Cardiovascular disease (CVD), especially coronary heart disease (CHD) and stroke, is the leading killer in Western Society and its prevalence is increasing dramatically in developing nations [1].

Atherothrombotic disease is the consequence of conventional risk factors such as smoking, hypertension, hyperlipidemia, insulin resistance and diabetes, and obesity. Novel risk factors include highly sensitive C-reactive protein (hs-CRP) and other markers of inflammation, homocysteine, and lipoprotein (a) [2].

Along with genetic factors and age, lifestyle and diet are also considered important risk factors. Reduction in dietary consumption of animal fat, cholesterol, and sodium should be the mainstay of population-wide CHD prevention [3]. Dietary interventions should be the initial step in the treatment of CVD. In particular, a group of phytochemical substances in carotenoids which is responsible for the color of food play an important role in the prevention of human diseases and the maintenance of good health [4].

## 2. Oxidative Stress and Antioxidants

Oxidative modification of low density lipoprotein cholesterol (LDL-C) play an important role in the initiation and progression of the atherosclerotic process, a *continuum pathophysiological process* that includes oxidative stress, endothelial dysfunction, inflammatory process, and vascular remodelling [5].

Specifically, CVD are associated with increased production of reactive oxygen species (ROS) and a compromised endogenous anti-oxidant defense system. Oxidative stress is tightly regulated by a balance between production and removal of ROS [6], which are natural by-products of metabolism with important roles in cell signaling. However, excessive levels of ROS can be toxic to cells, *i.e.*, whenever the expression of anti-oxidant enzymes, including superoxide dismutases (SODs), heme oxygenase-1 (HO-1), NAD(P)H quinine oxidoreductase-1 (NQO-1), catalase and thioredoxin are not sufficient to control ROS and minimize ROS-induced damage [7]. A compromised anti-oxidant defense system can lead to excessive oxidative stress and ultimately result in cell damage [8].

Numerous studies indicate that increased oxidative stress may be involved in the pathogenesis of CVD. Several animal models suggest that when endogenous anti-oxidant systems are overwhelmed, exogenous antioxidant supplementation can be used for preventive and/or therapeutic intervention of oxidative cardiovascular disorders [9]. In particular, SODs, catalase and glutathione peroxidase (GSH_Px_) are endogenous natural antioxidants present within human cells. In addition, antioxidants such as vitamin E, vitamin C, polyphenols and carotenoids are available from foods [10].

Current dietary guidelines to combat chronic diseases, including cancer and CHD, recommend increased intake of plant foods, including fruits and vegetables, which are rich sources of antioxidants [11]. The role of such dietary antioxidants in disease prevention has received much attention recently and appears to have a wide range of antiatherogenic properties [12,13]. These observations may explain the epidemiological data indicating that diets rich in fruits and vegetables are associated with a reduced risk of numerous chronic diseases [14].

Carotenoids are ubiquitous in nature and present in plants, algae and microorganisms. However, humans and other animals are unable to manufacture carotenoids and hence require these in their diet. There are two class types of carotenoids based on their chemical composition: carotenes and xanthopylls [15]. Astaxanthin and fucoxanthin are major marine carotenoids. Both these carotenoids show strong antioxidant activity which is attributed to quenching singlet oxygen and scavenging free radicals (FRs) [16].

## 3. Astaxanthin

### 3.1. Chemical Structure and Mechanism of Action

Astaxanthin is a xanthophyll carotenoid which contains two additional oxygenated groups on each ring structure compared with other carotenoids, resulting in enhanced antioxidant properties, approved in the 1999 as a dietary supplement by Food and Drug Amninistration. This compound occurs naturally in a wide variety of living organisms including microalgae (*Haematococcus pluvialis*, *Chlorella zofingiensis*, and *Chlorococcum* sp.), fungi (red yeast *Phaffia rhodozyma*), complex plants, seafood, and some birds such as flamingos and quail; it is reddish-coloured, and gives salmon, shrimp and lobster their distinctive colouration [17]. The microalga *H. pluvialis* has the highest capacity to accumulate astaxanthin up to 4–5% of cell dry weight. Astaxanthin has been attributed with extraordinary potential for protecting the organism against a wide range of diseases, and has considerable potential and promising applications in the prevention and treatment of various diseases, such as cancers, chronic inflammatory diseases, metabolic syndrome, diabetes, diabetic nephropathy, CVD, gastrointestinal and liver diseases, and neurodegenerative diseases [18].

Astaxanthin cannot be manufactured in animals or converted to vitamin A and therefore must be consumed in the diet. Xanthophyll carotenoids such as astaxanthin and canthaxanthin have antioxidant activity, are free radical scavengers, potent quenchers of ROS and nitrogen oxygen species (NOS), and chain-breaking antioxidants. They are superior antioxidants and scavengers of free radicals (FRs) compared to the carotenoids such as β-carotene [19].

Available forms of astaxanthin are represented by natural forms on an industrial scale of production [20,21]. The disodium disuccinate astaxanthin (DDA), a synthetic form of astaxanthin, overcame the limitations of caroteniods related to their poor aqueous solubility and enabled investigation of this agent in the animal models of myocardial ischaemia and reperfusion using both intravenous and oral routes of administration [22]. DDA has been shown to be very effective in animal cardiovascular studies administered both intravenously and orally [23–25].

### 3.2. Astaxanthin and Cardiovascular Disease: Experimental Studies

Astaxanthin has undergone investigation in a large number of experimental studies related to the cardiovascular and cerebrovascular systems. Only few studies have investigated the potential benefits of astaxanthin in human health and disease and most of these have been performed in healthy volunteers to assess dosing [26], bioavailability [27,28], safety [29], oxidative stress, and inflammation [26,30].

Studies conducted in healthy human volunteers have found significant reductions in oxidative stress, hyperlipidemia and inflammatory markers after astaxanthin oral supplementation. In particular, Cicero *et al.* [31] have demonstrated that food supplements for four weeks containing a combination of natural products such as berberine, policosanol, red yeast extracts, folic acid and astaxanthin could be a useful support to diet and life style changes to correct dyslipidemias and to reduce cardiovascular (CV) risk in 40 subjects with moderate mixed dyslipidemias. In this study total cholesterol (TC), LDL-C, high density lipoprotein cholesterol (HDL-C), non HDL-C, Apo-B, Apo-A, Lp(a) and triglycerides (TG) were measured before and at the end of treatments. Berberine and combinations of natural products (policosanol, red yeast extracts, folic acid and astaxanthin) significantly reduced TC (respectively by 16% and 20%), LDL-C (by 20% and 25%), Apo-B (by 15% and 29%) and TG (by 22% and 26%), and increased HDL-C (by 6.6% and 5.1%). Even Yoshida *et al.* [32] demonstrated in a randomized, placebo-controlled human study (61 non-obese subjects aged 20–65 years) that astaxanthin consumption (0, 6, 12, 18 mg/day for 12 weeks) ameliorates TG and HDL-C in correlation with increased adiponectin in humans. In this study, before and after tests, body mass index (BMI) and LDL-C were unaffected at all doses, however, TG decreased, while HDL-C increased significantly. Multiple comparison tests showed that 12 and 18 mg/day doses significantly reduced TG, and 6 and 12 mg doses significantly increased HDL-C. Serum adiponectin was increased by astaxanthin (12 and 18 mg/day), and changes of adiponectin correlated positively with HDL-C changes independent of age and BMI. Iwamoto *et al.* [26] demonstrated a significant inhibition of LDL-C oxidation in 24 healthy volunteers who took doses of astaxanthin (1.8, 3.6, 14.4 and 21.6 mg/day for 2 weeks). Even Karppi *et al.* [30] in a 12-week randomized double-blind study involving 40 healthy non-smoking Finnish males assessed a significant plasma reduction levels of 12- and 15-hydroxy fatty acids in those taking astaxanthin (8 mg/day) suggesting an important reduced fatty acid oxidation due to astaxanthin.

## 4. Fucoxanthin

### 4.1. Chemical Structure and Mechanism of Action

Fucoxanthin is a naturally occurring brown pigment that belongs to the class of non-provitamin A carotenoids, a class of 40-carbon organic molecules that consist of two groups: xanthophylls if their structure contains oxygen, and carotenes if there is no oxygen in their chemical formula. Fucoxanthin is a xanthophyll whose distinct structure includes an unusual allenic bond, epoxide group and conjugated carbonyl group in polyene chain [33] with antioxidant properties [34]. The difference, however is that fucoxanthin acts as an antioxidant under anoxic conditions whereas other carotenoids have practically no quenching abilities. Most tissues under physiological conditions have low oxygen presence. Furthermore, the typical antioxidants are usually proton donors (ascorbic acid, α-tocopherol, glutathione). Fucoxanthin, on the other hand, donates electron as a part of its free-radical quenching function. A combination of these distinct properties is very rarely found among naturally occurring food-derived compounds [35,36]. During normal metabolism the body produces heat. Fucoxanthin increases the amount of energy released as heat in fat tissue, a process also called thermogenesis. A published study reports that fucoxanthin affects multiple enzymes involved in fat metabolism causing an increase in the production of energy from fat [37].

### 4.2. Fucoxantin and Cardiovascular Disease: Experimental Studies (Human and Animal)

Experiments on stroke-prone spontaneously hypertensive rats (SHRSP) show the possible protective role of fucoxanthin in CVD. Thirty-three male SHRSP rats, 5 weeks of age, were purchased from the Disease Model Cooperative Research Association (Kyoto, Japan). Animals were divided into three groups: (1) kaolin group, which was given a normal diet [5% (w/w) kaolin, a non-nutrient material]; (2) Wakame (Undaria Pinnatifida) group [normal diet containing 5% (w/w) Wakame powder]; and (3) cellulose group [normal diet containing 5% (w/w) cellulose]. In this study, Wakame delayed the incidence of stroke signs and increased the life span of SHRSP. Wakame did not attenuate the development of hypertension in SHRSP [38].

Thrombosis is a major complication of coronary atherosclerosis that can lead to myocardial infarction. Docosahexaenoic acid (DHA) inhibits the synthesis of thromboxane A_2_ (TxA_2_) from arachidonic acid (AA) in platelets [39]. In addition, DHA enhances the production of prostacyclin, a prostaglandin that produces vasodilation and less sticky platelets [40]. Also epidemiologic and clinical trials demonstrated that fish oil such as eicosapentaenoic acid (EPA) and DHA, decreased LDL-C, TG and increased HDL-C concentrations. DHA content in fish oil fed to experimental animals inhibits the development of atherosclerosis, so the fucoxanthin may have an potential role in the modulation and prevention of human diseases, particularly to reducing the incidence of CVD [41,42].

### 4.3. Fucoxantin and Metabolic Syndrome: Experimental Studies (Human and Animal)

Among marine carotenoids, attention has been paid towards fucoxanthin in recent years, that is actually used for the treatment of methabolic syndrome and obesity [43], two important risk factors of CVD. Fucoxanthin has a unique structure including an allenic bond and 5,6-monoepoxide in the molecole, is a major carotenoid found in edible sceaweeds such as *Undaria pinnatifida*, *Hijikia fusiformis* and *Sargassum fulvelum* [44]. This molecule is under study for possible application in the fight against overweight and obesity since it promotes the reduction of abdominal fat. In this regard it is interesting to note an increased concentration of fat in the abdomen statistically correlated with an increased risk of CVD. A study conducted by Maeda *et al.* [43] in male Wister rats and female KK-Ay mice under different experimental diets (soybean oil, Undaria lipids, Undaria glycolipid fraction, crude fucoxanthin and purified fucoxanthin fed to different concentration according to their groups) for 4 weeks shows that Undaria lipids (containing 9.6% fucoxanthin) reduced significantly the weights of abdominal white adipose tissue (WAT) of both rats and mice. Body weights of mice fed Undaria lipid was significantly lower than that of controls [45]. Animal studies by one group of researchers suggest that fucoxanthin might prevent the growth of fat tissue and reduce abdominal fat, has a beneficial effects in stroke prevention, reduction of inflammation, and slowing the growth of various cancer cell type [38,45]. Woo *et al.* [37] in a recent study shows as, the fucoxanthin facilitates youthful energy metabolism by activating a special cellular mitochondrial protein called UCP-1, which induces the thermogenesis. This is important because obesity markedly increases the risk of CVD. Fucoxanthin has been found to reduce blood glucose in animals with diabetes and in normal mice that are fed high fat diets [46]. It appears that fucoxanthin is capable of upregulating glucose transporter, mRNA expression of L6 myotubes which are responsible for glucose transport in adult muscle tissue [47]. An interesting, extra, metabolic benefit of fucoxanthin administration in rodents is the promotion of the synthesis of DHA in the liver [43].

Because the metabolic syndrome is a collection of risk factors that substantially increase the chances of damage in the CVS, which can lead to a heart attack or stroke, the importance of the fucoxanthin in the regular metabolic syndrome would be very important to prevent CV damage. Clinical research also indicated that the metabolic boost from taking fucoxanthin did not stimulate the central nervous system, meaning it did not cause the jitters or lost sleep like caffeine, nicotine, or thyroid hormones. Only one study has currently been conducted in humans which has evaluated the effectiveness of fucoxanthin supplementation for weight loss. This study reports that the supplement, Xanthigen, which contains 300 mg pomegranate seed oil and 300 mg brown marine algae fucoxanthin significantly increased weight loss and reduced body and liver fats content in obese women treated for 16 weeks [48].

Fucoxanthin proved safe with no side effects, and even provided other health benefits, including improved cardiovascular health, reduction of inflammation (a major cause of heart disease), healthy cholesterol and TG levels, improvements in blood pressure levels, and healthy liver function [49–51].

## 5. Conclusion

Oxidative stress and inflammation play an important role in the pathophysiology of many chronic diseases including CVD [52]. The xanthophyll carotenoid dietary supplement, astaxanthin, has demonstrated to be a potential antioxidant and anti-inflammatory therapeutic agent in models of CVD. There have been human clinical studies using astaxanthin to assess its safety, bioavailability and clinical aspects relevant to oxidative stress and inflammation in CVS. There were no adverse effects reported. These demonstrated reduced markers of oxidative stress and inflammation and improved blood rheology. Astaxanthin has great potential as a potent antioxidant to be tested in human clinical trials based on theoretical grounds related to its physicochemical properties and on the basis of exciting preliminary experimental studies in CV models. Although its use in human clinical studies has been limited, so far no safety concerns have arisen [53]. We predict that because of its greater antioxidant potency and membrane preservation, astaxanthin will reduce measures of oxidative stress and inflammation and provide vascular benefits [54].

The versatile effects of fucoxanthin on intermediate metabolism make this carotenoid of great potential value in the prevention or management of the metabolic syndrome and obesity. The animal experiments with fucoxanthin stimulated researchers to recommend human clinical trials with fucoxanthin. As a carotenoid, fucoxanthin is a powerful antioxidant that protects cells from FRs damage. Future clinical studies and trials will help determine the efficacy of these marine carotenoids (asthaxantin and fucoxanthin) on vascular structure, function, oxidative stress and inflammation in a variety of patients at risk of, or with established CVD. These may lead to large interventional trials assessing CV morbidity and mortality.
